# Unlocking the power of precision medicine for pediatric low-grade gliomas: molecular characterization for targeted therapies with enhanced safety and efficacy

**DOI:** 10.3389/fonc.2023.1204829

**Published:** 2023-06-15

**Authors:** Selene Cipri, Giada Del Baldo, Francesco Fabozzi, Luigi Boccuto, Andrea Carai, Angela Mastronuzzi

**Affiliations:** ^1^ Department of Hematology/Oncology, Cell Therapy, Gene Therapies and Hemopoietic Transplant, Bambino Gesù Children’s Hospital, IRCCS, Rome, Italy; ^2^ Department of Experimental Medicine, Sapienza University of Rome, Rome, Italy; ^3^ Healthcare Genetics Program, School of Nursing, College of Behavioral, Social and Health Sciences, Clemson University, Clemson, SC, United States; ^4^ Department of Neurosciences, Neurosurgery Unit, Bambino Gesù Children’s Hospital, IRCCS, Rome, Italy

**Keywords:** pediatric low-grade glioma, brain tumors, neuro-oncology, molecular diagnostic, clinical trials, targeted therapies, risk stratification, glioma

## Abstract

In the past decade significant advancements have been made in the discovery of targetable lesions in pediatric low-grade gliomas (pLGGs). These tumors account for 30-50% of all pediatric brain tumors with generally a favorable prognosis. The latest 2021 WHO classification of pLGGs places a strong emphasis on molecular characterization for significant implications on prognosis, diagnosis, management, and the potential target treatment. With the technological advances and new applications in molecular diagnostics, the molecular characterization of pLGGs has revealed that tumors that appear similar under a microscope can have different genetic and molecular characteristics. Therefore, the new classification system divides pLGGs into several distinct subtypes based on these characteristics, enabling a more accurate strategy for diagnosis and personalized therapy based on the specific genetic and molecular abnormalities present in each tumor. This approach holds great promise for improving outcomes for patients with pLGGs, highlighting the importance of the recent breakthroughs in the discovery of targetable lesions.

## 1 Introduction

Pediatric low-grade gliomas (pLGGs) are one of the more frequent pediatric brain tumors accounting for about 30-50% of central nervous system (CNS) tumors of pediatric patients. They carry a favorable prognosis with an overall survival (OS) at 10 years greater than 90%. In a minority of cases an aggressive behaviour is described (1, 2).

To date, complete resection is the most favourable outcome measurement of the patients, but it is not easy to conduct for deep or infiltrative lesions (3), and for progressive residual disease adjuvant chemotherapy or radiation were historically performed (4–12). However, we did not forget that the side effects are far from negligible (5, 13–16). Pediatric LGGs comprise of a heterogeneous group of tumors, and recently molecular studies led to a better clarification and classification of pLGGs, and which paved the way for promising new therapeutic strategies.

Many types of tumors are included under the umbrella of pLGGs. Historically, these types of neoplasms have been classified on the basis of histology, but today we know that the same histologies can underlie different entities and histological classification alone is no longer useful (17). The molecular characterization advancements have revealed that appear similar under a microscope can have different genetic and molecular characteristics, so the new classification system divides pLGGs into several distinct subtypes based on these characteristics, rather than solely on their histological appearance. Better knowledge of the molecular characteristics, technological advances, and new applications in molecular diagnostics of pLGGs have helped overcome these challenges (18).

The updated 2021 World Health Organization (WHO) Classification of Tumors of the CNS has reflected the focus on the integration of histopathological and molecular characteristics to facilitate a more accurate diagnosis (19). In the new classification of pLGGs places a strong emphasis on the molecular characterization of these tumors for significant implications on the prognosis, diagnosis, management, and finally development of personalized treatment (19).

This classification describes three families of tumors that encompass pLGGs and glioneuronal tumors (GNTs) (**Table 1**), which are now defined by their driver molecular alterations rather than by histopathological features alone: “Glioneuronal and neuronal tumor”, “Circumscribed astrocytic gliomas” and “Pediatric type diffuse low-grade gliomas” (17, 19).

**Table 1:** WHO 2021 classification for pLGG/low-grade GNTs (19).

Pediatric-type diffuse low-grade gliomas	Circumscribed astrocytic gliomas	Glioneuronal and neuronal tumors
1. Diffuse astrocytoma, MYB- or MYBL1-altered 2. Angiocentric glioma 3. Polymorphous low-grade neuroepithelial tumor of the young (PLNTY) 4. Diffuse low-grade glioma, MAPK pathway-altered	1. Pilocytic astrocytoma 2. Pleomorphic xanthoastrocytoma (PXA) 3. Subependymal giant cell astrocytoma (SEGA) 4. Choroid glioma	1. Ganglioglioma 2. Desmoplastic infantile ganglioglioma/desmoplastic infantile astrocytoma 3. Dysembryoplastic neuroepithelial tumor 4. Diffuse glioneuronal tumor with oligodendroglioma-like features and nuclear clusters 5. Rosette-forming glioneuronal tumor 6. Papillary glioneuronal tumor 7. Myxoid glioneuronal tumor 8. Diffuse leptomeningeal glioneuronal tumor (DLGNT) 9. Gangliocytoma 10. Multinodular and vacuolating neuronal tumor 11. Dysplastic cerebellar gangliocytoma (Lhermitte-Duclos disease) 12. Central neurocytoma 13. Extraventricular neurocytoma 14. Cerebellar liponeurocytoma

In this review, we described the major molecular alterations detected in pLGGs and the molecular target therapy available to date.

## 2 MAPK/ERK and PI3K/AKT/mTOR signaling pathway alterations in pediatric low-grade gliomas

### 2.1 MAPK/ERK and PI3K/AKT/mTOR signaling pathway in physiological conditions

**Figure 1:**
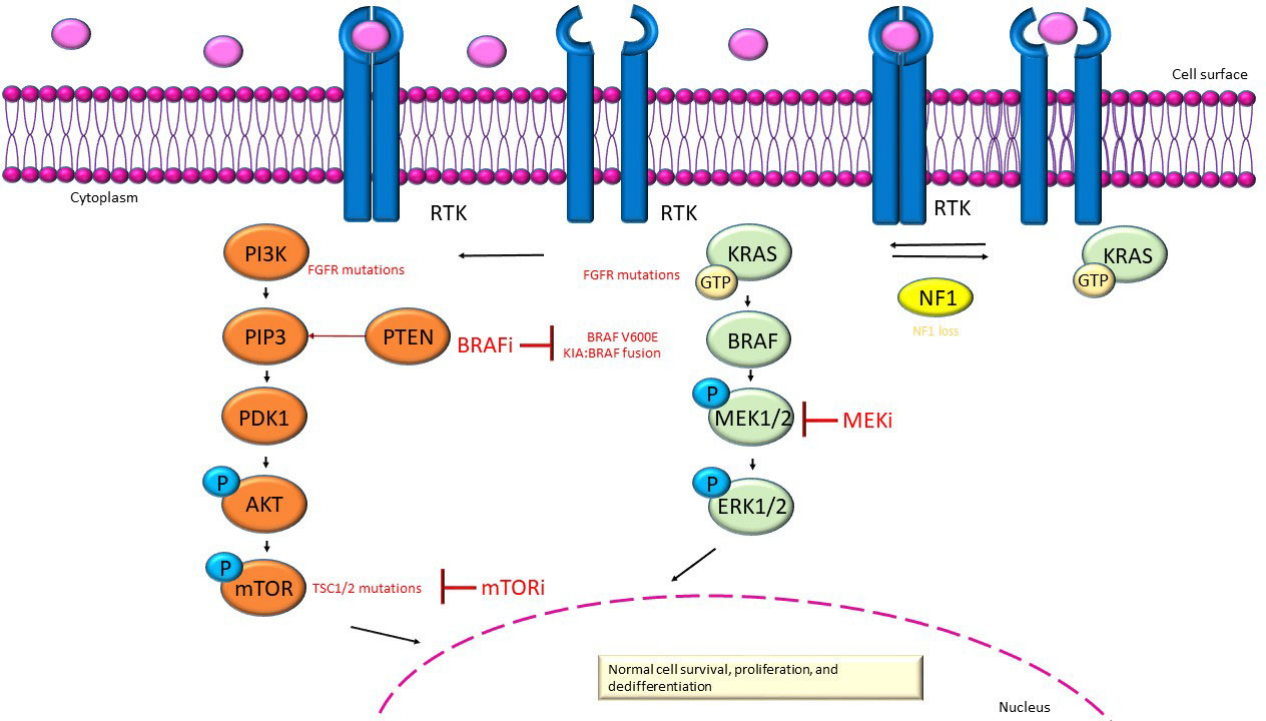
MAPK/ERK (green) and PI3K/AKT/mTOR (orange) signaling pathway. In red show the inhibitors and the alterations in gene that are causative to dysregulation of these pathways in pLGGs.

In the Mitogen-Activated Protein Kinase/extracellular signal-regulated kinases (MAPK/ERK) signaling pathway (**Figure 1**), stimulation of receptor tyrosine kinases (RTKs) in physiological conditions causes MAPK activation. The activation of Ras enabled the activity of the serine/threonine-protein kinase B-raf, which homodimerizes or heterodimerizes by phosphorylating and triggering mitogen-activated protein kinase kinase (MEK1 and MEK2), which in turn phosphorylates and trigger ERK 1 and ERK2. Finally, the latter boost dedifferentiation, proliferation and cell survival by scalable transcriptional asset within the nucleus; consequently, downstream activation of ERK causes feedback inhibition of the upstream pathway (20–23).

The activation of the phosphatidylinositol 3-kinase/protein kinase B/mechanistic target of rapamycin (PI3K/AKT/mTOR) pathway (**Figure 1**) is mediated by transmembrane receptor tyrosine kinases of growth factors (24). The Phosphatidylinositol 3-kinase (PI3K) is triggered from the bond of oncogenes or growth factors (24). PI3K transfom phosphatidylinositol-4,5-phosphate (PIP2) to phosphatidylinositol-3,4,5-phosphate (PIP3) (24). The lipid Phosphatase and tensin homolog (PTEN) has the function of countering the build-up of PIP3 and enroll to the membrane protein kinase B (PKB or Akt) and phosphoinositide-dependent kinase 1 (PDK1), which are phosphorylated and triggered (24). The molecular complexes mTOR Complex 1 (mTORC1) and mTOR Complex 2 (mTORC2) have both a catalytic subunit mTOR which is negatively regulates by the heterodimer of tuberous sclerosis proteins TSC1 (hamartin) and TSC2 (tuberin) [a GTPase-activating complex (GAP) to Rheb (homolog of Ras enriched in the brain)], in contrast the activation of the PI3K pathway, AKT phosphoryl TSC2 and disable the TSC1/TSC2 complex (25–27). Mechanistic target of rapamycin (mTOR) can even be triggered by the MAPK pathway via RAS/MEK/ERK (28).The phosphorylation of TSC2 by ERK and ribosomal S6 kinase (RSK) can induce mTORC1 activation; instead, RSK can target the mTORC1 complex by directly promoting the kinase activity of the complex (28). Aberrant activation of mTOR may be related to various mutations that activate the mTOR pathway, such as alterations at mTOR negative regulators or mTOR pathway components (28). PI3K activation facilitates the activation of mTORC1 and mTORC2. Activation of mTORC1 downstream of PI3K and protein- kinase B (AKT) promotes cell survival, growth and proliferation. Moreover, mTORC2 increases cell proliferation and survival through regulation of protein kinases, including AKT, which provides significant motivation for further studies on therapeutic targeting of mTOR complexes in cancer, as mTOR plays an important role in tumor progression (29).

### 2.2 *BRAF* alterations and targeted therapy

Within pLGG a notorious troublemaker has been identified: the B-Raf proto-oncogene, serine/threonine kinase (*BRAF*) gene. This gene encodes a protein from the RAF family that is responsible for regulating the MAPK/ERK pathway (30). Pediatric LGGs often harbor alterations in the BRAF gene, such as the p.V600E point mutation and the translocation between *BRAF* and *KIAA1549*. These alterations result in a hyperactive protein that wreaks havoc on the MAPK pathway, leading to uncontrolled cell division and tumorigenesis (31–41).

Most of sporadic pLGGs are characterize by *BRAF* mutations (2, 42). A three-class system was defined based on the result of *BRAF* mutations on the activity of the encoded protein. RAS-independent as monomers represent the class I mutations RAS-independent as dimers belong to class II mutations, and RAS-dependent with altered kinase activity are class III mutations (24).

Class I mutations, which include the mutation on 600 codon of *BRAF*, hyperactivate kinases through promotion of MEK/ERK activation regardless of the protein dimerization (for example with Raf has low effect) and activation of RAS (24). In fact, inhibition of upstream ERK feedback has any impact on class I mutations because, although *BRAF* p.V600E dimerization stays Ras dependent and is blocked by upstream ERK response, but it can yet turn on the pathway like monomer (43, 44). A point mutation c.1799T>A causes the replacement of valine with glutamic acid at codon 600 (p.V600E) within the gene’s activation region. The occurrence of *BRAF* p.V600E in non-pilocytic pLGGs varies significantly depending on the tumor’s histology and location. Ganglioglioma (25-45%) and pleomorphic xanthoastrocytomas (40-80%) frequently exhibit the variant, while it is less commonly observed in pilocytic astrocytoma (PA) (5-10%) and GNTs (5%) (45–52). Combining histological and molecular data helps to achieve a more precise diagnosis. For example, identifying *BRAF* p.V600E, along with the detection of a mildly and minimally atypical glial proliferation without eosinophilic granular bodies and Rosenthal fibers (RFs), enables categorizing the tumor as a “low-grade diffuse glioma” (19). In a retrospective study, 17% of children with LGGs carried the BRAF p.V600E variant and presented a 10-year progression-free survival (PFS) rate of about 27% versus a 60% rate for those without the same variant (53). This trend is confirmed by several studies (53–57). However, almost one-third of patients who experienced complete resection relapsed, indicating that *BRAF* p.V600E is the most interfering phenotype than other mutation known in patients with pLGGs (53). A progression-free survival of 5-year are reported in a study on children with low diencephalic astrocytomas carried *BRAF* p.V600E (22%) versus the children without BRAF mutations (52%) (58). The *BRAF* p.V600E variants were more frequently detected in pLGGs that transform into high-grade gliomas (59). Several studies have demonstrated that 25% of patients with pLGGs exhibit BRAF p.V600E in conjunction with deletions of *CDKN2A*, which probably operates as a second hit, altering the regulation of cell cycle (38, 45, 53, 60–62). Tumors with *BRAF* p.V600E and a *CDKN2A* deletion represent a separate subtype of pLGGs which are inclined to change into HGG (59). Reports show that both these mutations are related with oncogene-induced senescence escape and poorer OS and PFS (38, 45, 62). Therefore, pLGGs with *CDKN2A* deletions, particularly those with p.V600E or possible high-grade histological characteristics, should be considered high-risk tumors requiring close clinical follow-up (63). Finally, some studies have reported rare cases of *BRAF* missense variants at the p.V600 residue, in which valine is replaced with other amino acids such as lysine (p.V600K), aspartic acid (p.V600D), or arginine (p.V600R). Desmoplastic infant astrocytomas/gliomas exhibit the p.V600K variant, while the BRAF p.V504_R506dup variant was reported in cases with PA. Supratentorial lesions are more frequently associated with *BRAF* p.V600E, while cerebellar lesions more commonly present *KIAA1549-BRAF* (47, 51, 64).

Class II mutations involve *BRAF-KIAA1549* fusion and other gene fusions. They trigger both intermediate and high kinase activity, requiring dimerization of the protein to activate the MEK/ERK pathway (24). The *KIAA1549-BRAF* fusion is a great slice of gene fusions involving BRAF in pLGGs, accounting for a whopping 30-40% of cases (65). The *KIAA1549* gene belongs to the mysterious UPF0606 family and we are still trying to understand what it does (66). *KIAA1549-BRAF* fusion is a major player in a variety of CNS tumors. It is particularly prevalent in infratentorial and midline PAs, although it shows up less often in supratentorial tumors (34, 38, 67–72). Interestingly, early studies have proven that fusions that involved these genes are correlated to tandem duplication that creates a brand-new oncogenic fusion. This rearrangement messes with domain at the N-terminal regulatory region of the BRAF protein, which in turn causes RAS/MAPK pathway altered regulation (35, 36, 73). But despite these complicated genetics, one thing is clear: the presence of the *KIAA1549-BRAF* fusion is associated with better OS and PFS in pLGGs that cannot be fully removed and do not tend to progress too quickly (53, 69, 70, 72). Unfortunately, in cases where the tumor is located in a difficult-to-reach part of the brain, progression is more likely (53).

Other alterations in addition to the *BRAF-KIAA1549* fusion, such as *CDKN2A* deletions, and tumor location may alter the outcome of the patient (45, 74).

Other rearrangements that activate the RAS/MAPK pathway and involving *BRAF* are the *MKRN1* (Makorin Ring Finger Protein 1)*, SRGAP* (SLIT-ROBO Rho GTPase Activating Protein 2)*, GIT2* (GIT ArfGAP 2)*, FAM131B* (Family with Sequence Similarity 131 Member B)*, RNF130* (Ring Finger Protein 130)*, CLCN6* (Chloride Voltage-Gated Channel 6)*, GNAI1* (G Protein Subunit Alpha I1), and *FXR1* (FMR1 Autosomal Homolog 1) mergers involving deletion of BRAF N-regulatory domain (34, 74–76). These non-canonical fusions in particular manifest in older children and adolescents, frequently in brainstem lesions and hemispheres, and are also observed in a series of rare histological profiles (67, 75–77).

Class III mutations are found to be linked to poor or no kinase activity and need both the activation of upstream RAS and dimerization with CRAF to further induce induction of MER/ERK pathway activation (24). In literature are reported a few cases of *BRAF* p.D594G and p.G466V mutations (78).

Finally, *BRAF* p.V600E mutations and *BRAF* fusions enable molecular characterization of nearly 2/3 of pLGGs (2).

BRAF inhibitors (BRAFi), including vemurafenib, dabrafenib, and encorafenib, are drugs that selectively bind to mutated B-Raf proteins and block the activation of MEK by inhibiting the MAPK/ERK cascade signaling (79). Clinical studies have demonstrated that vemurafenib and dabrafenib first-generation BRAFi are highlyeffective in treating children with LGGs, with numerous case reports showing complete responses (52, 80–89). However, these inhibitors have been found to activate the signaling pathway of RAS/MAPK when used in tumors with the fusions that involved KIAA1549 and BRAF or BRAF *wild-type* (wt) (90, 91). To address this issue, “paradox-breaker” secondo generation agents have been developed that do not activate the RAS/MAPK pathway (92). Ongoing clinical trials are investigating the use of the dual combination of BRAFi and MEK inhibitors (MEKi) to treat BRAF p.V600 mutation-positive gliomas (**Table 2**) (93–98). There are also emerging new class II BRAF inhibitors, such as TAK-580, that look promising in treating LGGs (116). Overall, BRAF inhibitors offer a remarkable therapeutic option for pLGGs, particularly in pediatric patients where traditional treatment methods may have long-term effects on brain development.

**Table 2:** List of clinical trials for pLGG using targeted therapy.

Drug	Trial ID	Phase	Target	Information	Reference
BRAF Inhibitors	NCT02684058	Phase II	Children and Adolescent Patients With BRAF V600 Mutation Positive Low-Grade Glioma (LGG) or Relapsed or Refractory High-Grade Glioma (HGG)	Pediatric Study With Dabrafenib in Combination With Trametinib in Patients with HGG and LGG	(93)
	NCT01748149	Early Phase I	Children with recurrent or refractory gliomas containing the BRAFV600E or BRAF Ins T mutation	Vemurafenib in Children With Recurrent/Refractory Gliomas	(94)
	NCT03429803	Phase I	/	This research study on the drug Tovorafenib/DAY101 (formerly TAK-580, MLN2480) as a possible treatment a low-grade glioma that has not responded to other treatments	(95)
	NCT02428712	Phase II	Adolescent patients with advanced BRAF- mutated tumors	A Study of FORE8394 as a Single Agent in Patients With Advanced Unresectable Solid Tumors	(96)
	NCT01677741	Phase I/IIa	Children and Adolescent Subjects With Advanced BRAF V600-Mutation Positive Solid Tumors	A Study to Determine Safety, Tolerability and Pharmacokinetics of Oral Dabrafenib In Children and Adolescent Subjects	(97)
	NCT02034110	Phase II	BRAFV600E mutation	Efficacy and Safety of the Combination Therapy of Dabrafenib and Trametinib in Subjects With BRAF V600E- Mutated Rare Cancers	(98)
MEK Inhibitors	NCT01089101	Phase II	Presence or absence of BRAF V600E mutations or BRAF KIAA1549 fusion	Selumetinib in Treating Young Patients With Recurrent or Refractory Low-Grade Glioma	(99)
	NCT03363217	Phase II	NF1 LGG with KIAA 1549-BRAF fusion -Progressing-refractory glioma with activation of the MPAK/ERK pathway who do not meet criteria for other study groups	Trametinib for Pediatric Neuro-oncology Patients With Refractory Tumor and Activation of the MAPK/ERK Pathway	(100)
	NCT02639546	Phase I/II	/	Safety and Pharmacokinetics of Cobimetinib in Pediatric and Young Adult Participants With Previously Treated Solid Tumors (iMATRIXcobi)	(101)
	NCT02285439	Phase I/II	Children with LGG characterized by a BRAF truncated fusion (KIAA1549 and similar translocations) Children with NF1 and LGG Children with tumors involving the Ras/Raf pathway not included in strata 1 or 2	Study of MEK162 for Children With Low-Grade Gliomas	(102)
	NCT03871257	Phase III	Patients must have neurofibromatosis type 1 (NF1) based on clinical criteria and/or germline genetic testing * Patients must be newly diagnosed or have previously diagnosed NF-1 associated LGG that has not been treated with any modality other than surgery	A Study of the Drugs Selumetinib Versus Carboplatin/Vincristine in Patients With Neurofibromatosis and Low-Grade Glioma	(103)
	NCT04166409	Phase III	Newly Diagnosed or Previously Untreated Low-Grade Glioma (LGG) Not Associated With BRAFV600E Mutations or Systemic Neurofibromatosis Type 1 (NF1)	A Study of the Drugs Selumetinib vs. Carboplatin and Vincristine in Patients With Low-Grade Glioma	(104)
	NCT04576117	Phase III	Patients with BRAF rearranged LGG and patients with non-BRAF rearranged LGG	A Study to Compare Treatment With the Drug Selumetinib Alone Versus Selumetinib and Vinblastine in Patients With Recurrent or Progressive Low-Grade Glioma	(105)
	NCT04201457	Phase I/II	o LGG with BRAF V600E/D/K mutation; o LGG with BRAF duplication or fusion with any partner or LGG with NF1.	A Trial of Dabrafenib, Trametinib and Hydroxychloroquine for Patients With Recurrent LGG or HGG With a BRAF Aberration	(106)
	NCT02124772	Phase I/II	Children and Adolescents Subjects With Cancer or Plexiform Neurofibromas and Trametinib in Combination With Dabrafenib in Children and Adolescents With Cancers Harboring V600 Mutations	Study to Investigate Safety, Pharmacokinetic (PK), Pharmacodynamic (PD) and Clinical Activity of Trametinib in Subjects With Cancer or Plexiform Neurofibromas and Trametinib in Combination With Dabrafenib in Subjects With Cancers Harboring V600 Mutations	(107)
	NCT04485559	Phase I	Participants with LGG who have had surgery alone are not eligible. Participants with neurofibromatosis type 1 (NF-1) are eligible but must have available tissue per study requirements neurofibromatosis (NF) status will be collected	Trametinib and Everolimus for Treatment of Pediatric and Young Adult Patients With Recurrent Gliomas (PNOC021)	(108)
	NCT05180825	Phase II	Patients with a determination of a negative BRAFv600 mutation by immunohistochemistry and/or molecular methods and patients without NF1	Pediatric Low Grade Glioma – MEK inhibitor TRIal vs Chemotherapy (PLGG - MEKTRIC)	(109)
	NCT03975829	Phase IV	Patients who received monotherapy of either of dabrafenib or trametinib Patients who received combination of dabrafenib and trametinib	Pediatric Long-Term Follow-up and Rollover Study	(110)
mTOR Inhibitors	NCT01158651	Phase II	Children with NF1 progressive LGG	Everolimus for Children With NF1 Chemotherapy-Refractory Radiographic Progressive Low Grade Gliomas (NFC-RAD001)	(111)
	NCT00782626	Phase II	Exclusion criteria: presence of NF1 by clinical examination or by genetic testing	Everolimus (RAD001) for Children With Chemotherapy-Refractory Progressive or Recurrent Low-Grade Gliomas	(112)
	NCT01734512	Phase II	/	PNOC 001: Phase II Study of Everolimus for Recurrent or Progressive Low-grade Gliomas in Children	(113)
NTRK Inhibitors	NCT02650401	Phase II	Primary brain tumors with NTRK1/2/3 or ROS1 gene fusions; gene fusions are defined as those predicted to translate into a fusion protein with a functional TRKA/B/C or ROS1 kinase domain, without a concomitant second oncodriver as determined by a nucleic acid-based diagnostic testing method Extracranial solid tumors (including NB) with NTRK1/2/3 or ROS1 gene fusions; gene fusions are defined as those predicted to translate into a fusion protein with a functional TRKA/B/C or ROS1 kinase domain, without a concomitant second oncodriver as determined by a nucleic acid-based diagnostic testing method	A Phase 1/2, Open-Label, Dose-Escalation And Expansion Study Of Entrectinib (Rxdx-101) In Pediatrics With Locally Advanced Or Metastatic Solid Or Primary CNS Tumors And/Or Who Have No Satisfactory Treatment Options	(114)
IDH1 Inhibitors	NCT04164901	Phase III	Patients (>/= 12 years) Residual or Recurrent Grade 2 Glioma with confirmed IDH1 (IDH1 R132H/C/G/S/L mutation variants tested) or IDH2 (IDH2 R172K/M/W/S/G mutation variants tested) gene mutation status disease	Study of Vorasidenib (AG-881) in Participants With Residual or Recurrent Grade 2 Glioma With an IDH1 or IDH2 Mutation (INDIGO)	(115)

In addition, MEKi have emerged as a potential treatment strategy for pLGG patients and ongoing clinical trials are examining the use of several drugs such as selumetinib in treating of young patients with recurrent or refractory LGGs (characterized by the presence or absence of *BRAF* V600E mutations or *BRAF-KIAA1549* fusion); trametinib for pediatric neuro-oncology patients with refractory tumor and activation of the MAPK/ERK pathway causative by a KIAA 1549-BRAF fusion; and a study of MEK162 for children with LGGs characterized by a *BRAF* truncated fusion (KIAA1549 and similar translocations) (**Table 2**) (99–102, 105, 108–110).

### 2.3 *FGFR1* alterations

The subunits of the RTKs, which are crucial in transmitting the MAPK signal, are encoded by genes pertaining to the Fibroblast Growth Factor Receptor (*FGFR*) family (*FGFR1-4*) (117). Fibroblast Growth Factor Receptor 1 (*FGFR1*) alterations are common in pLGGs (40, 64, 76), with p.N546K and p.K656E being the most frequent mutations observed in 5-10% of patients, while *FGFR1* TKD duplication is detected in 2-23% of tumors. *FGFR1* mutations have been identified in various pLGGs, including PA with an unfavorable prognosis, although none of these changes is histologically specific (64, 76, 118, 119). Fusion genes involving *FGFR*’s N-terminal domain and other genes such as *TACC1* (Transforming Acidic Coiled-Coil Containing Protein 1)*, KIAA1598* (Shootin 1)*, TACC2* (Transforming Acidic Coiled-Coil Containing Protein 2)*, TACC3* (Transforming Acidic Coiled-Coil Containing Protein 3) and *KIAA1598* (Shootin 1) characterize pLGGs (120).

All these changes lead to FGFR1 self-phosphorylation, are correlated to the up alteration of the RAS/MAPK pathway and PI3K/AKT/mTOR pathway (76). *FGFR1* alterations’ clinical manifestations are not yet fully understood and can be the product of more alterations in the genes that are mentioned earlier (64, 76, 119).

### 2.4 Other alterations in pLGGs and targeted therapy

The neurotrophic receptor of tyrosine kinase (NTRK) family and the *ALK* gene have significant roles in the development and fuctions of the CNS (81–88). In pLGGs, *NTRK* gene fusions including *NTRK1/2/3*, *SLMAP-NTRK2, TPM3-NTRK1, RBPMS-NTRK3* and *ETV6-NTRK3* are rare (64, 76, 121, 122). *ALK* alterations are also uncommon in pLGGs, but the fusions that involved *CCDC88A* and *PPP1CB* with *AKT* being the more prevalent and resulting from chromothripsis (123–125). These changes cause tumorigenesis by modifying the RAS/MAPK and PI3K/AKT/mTOR pathways via abnormal NTRK kinase domain dimerization or ectopic expression of the product fusion involved (125–129). Alterations in *NTRK* gene are rare in pLGGs, while they are common in adult cancers and this has enabled the development and testing of drugs already approved by the FDA. Entrectinib was approved for treatment of solid tumors when patients carrier a *NTRK* gene fusion and larotrectinib for both population of patients with solid tumors who carrier a fusion that involved TRK without a mutation known as related to acquired resistance, who are metastatic or in whom surgical excision may cause significant morbidity and who have no suitable treatment options or progressed after therapy (130–134).

In pediatric gliomas, in particular, both entrectinib and larotrectinib showed potent antitumor effects (135–137). These findings resulted to a phase I/II study presently ongoing in children to assess entrectinib in primary tumors of CNS (114).

To date there is a lack of data on the role of larotrectinib in primary CNS tumors, as few case reports have been published in particular on pediatric high-grade gliomas (pHGGs) and clinical trials have not yet been completed (138–143).

Finally, another rarely reported alteration in pLGGs, involves IDH1 whose role in these types of pediatric cancers is unclear to date (64, 144). A study of patients with LGGs and mutation in IDH1 found excellent short-term survival, but with a 5-year PFS of less than 43% and mortality after 10 years (145). To date, Vorasidenib (Ag-881), a new inhibitor against IDH1 and IDH2 mutation with high brain penetration, show a good results in clinical trial on adult patients with LGG (above/equal to 18 years of age) and IDH1 mutations (146–150). Consequently, Ag-881 was tested in a phase III clinical trial (INDIGO) in patients up/equal to 12 years of age and with residual or recurrent grade 2 Glioma who carried an IDH1 R132H/C/G/S/L or IDH2 mutation (115).

Another IDH1 inhibitor is FT-2102, used specifically in the treatment of myelodysplastic syndromes and AML, was tested in the adult population with solid tumors and gliomas in which mutation in IDH1 was found (151, 152). Other studies are on going in the adult population (153).

### 2.5 Cancer predisposition syndrome associated with pLGG: from alterations involving the RAS/MAPK and mTOR signaling pathway to targeted therapy

Alterations involving the RAS/MAPK pathway in pLGG pathogenesis have been studied in patients with Neurofibromatosis type 1 (NF1), of which 10-15% develop low-grade gliomas (154–156).

About 20% of patients with NF1 develop pLGGs (157): they often present with optically induced tumors that are not biopsied, NF1-pLGGs are asymptomatic and indolent, do not require any treatment, and in some cases regress without treatment; however, in case of clinical deterioration (more frequently vision loss), the first line of therapy used is chemotherapy (158–161). In addition, some studies have repositioned NF1-pLGGs patients with other additional genetic alterations of the RAS/MAPK pathway (162). Seventy-five percent of NF1-pLGGs carried a genetic mutation in one or more genes that are involved in biological process (162). Finally, in pLGGs involving *NF1*-associated alterations, *BRAF* variants are rare (68, 162).

Target therapies have also been attempted and described in patients with NF1. MEKi have emerged as a potential treatment strategy for pLGG patients who are unresponsive to BRAFi, such as those with *KIAA1549-BRAF* or NF1-pLGG (163). Ongoing clinical trials are exploring efficacy of treatment with selumetinib, trametinib, cobimetinib, and binimetinib in young patients with refractory pLGGs (**Table 2**) (99–102). Phase I/II trials on selumetinib have demonstrated its stability or reduction of tumor size in pediatric patients with NF1-associated and sporadic form of pLGGs, with similar results observed in a study of children with progressive/recurrent PA (**Table 2**) (164, 165). Phase III clinical trial are currently exploring the efficacy of selumetinib as a frontline therapy for both NF1-associated and NF1-non-associated pLGGs (**Table 2**) (103, 104).Trametinib and binimetinib have also shown promise in small studies, with trametinib appearing effective as a single drug or in compound with dabrafenib (107, 166–171). Broader studies are required to assess the tolerability of MEKi in pLGG patients (159). Overall, MEKi showed a promising therapeutic alternative for pLGGs, particularly for children with NF1-associated tumors without *BRAF* gene alterations (172).

of the vaste majority of children with tuberous sclerosis have a germline pathogenetic variant in tuberous sclerosis genes (*TSC1* or *TSC2*), that increase the risk of developing subependymal giant cell astrocytomas,subependymal nodules and cortical tubers, as some pathogenetic variants in these genes lead to mTOR pathway activation (173). Subependymal giant cell astrocytomas are led by mTOR activation; mTORi are active drugs that may induce the regression of the tumor in children affected by these tumors (173). Finally, germline mutations in genes (more than 10) involved in the RAS-MAPK pathway are causative of Noonan syndrome (NS), an autosomal dominant congenital condition (174). Noonan Syndrome is correlated to develop a brain tumors (174). Our group described 13-year-old patient with NS who developed a cerebellar PA, an optic pathway glioma (OPG) and a left temporal lobe glioneuronal neoplasm. A pathogenetic variant in the *PTPN11* gene was found and the molecular characterization of the GNT revealed elevated levels of phosphorylated mTOR (pMTOR) (175). Tyrosine phosphatase adaptor protein is encoded by *PTPN11* gene and it is involved, as reported before, in the RAS/MAPK pathway (78). Additionally, PTPN11 overexpression alone does not significantly activate the RAS/MAPK pathway, and further alterations like mutations in the FGFR1 gene, which activate the PI3K/AKT/mTOR pathway, are required (154).

mTOR was found to be excessively activated in pLGGs associated with syndromic conditions like TS and NF1 and this prompted to the support for the use of mTORi such as everolimus in clinical treatment alternative strategy (161, 176–181). Studies have highlighted that inhibiting the mTOR pathway is a promising therapeutic strategy for pLGG, and experimental evidence is emerging that suggests mTOR pathway activation may be a feature of most pLGGs (111, 173, 182–185). Everolimus has been successful in treating subependymal giant cell astrocytoma, a subtype of pLGG, and has demonstrated seizure control and tumor volume reduction in TS patients with SEGAs (173, 183, 186–188).

Our group suggested using everolimus for patients with RASopathies and brain tumors that have overactive mTOR signaling, and a phase II study is ongoing for recurrent or progressive pLGGs children. Everolimus has also been shown to provide a significant therapeutic alternative to immediate surgery in TSC patients, allowing for the postponement of a neurosurgical resection (**Table 2**) (112, 113, 175, 189). Moreover, we reported the first use of everolimus in children with pLGGs who were chemo- and radiotherapy-naïve (190). The results showed a lack of progression with a manageable toxicity profile, providing preliminary support for everolimus as a therapy for pLGG (190).

Overall, more studies are needed to develop innovative therapies for pLGG patients based on oncological mechanisms related to tumor development.

## 3 Discussion

Pediatric LGGs represent 30-50% of CNS childhood tumors; their prognosis greatly differed between tumor clusters and is dictated by a variety of factors, which include age at diagnosis, localization, and extension of resection surgery. pLGG represent a chronic disease and despite the treatments available to date, associated long-term morbidity remain of paramount importance.

Surgery is currently the standard of care, and children who undergo gross tumor resection (GTR) often do not demand additional action other than regularly follow-up. In a cohort of 518 patients, the 5-year PFS rate for children who have undergone GTR was high (94%) with an OS rate of 99%; any degree of residual tumor predicted a worse PFS, with up to 44% of patients with limited residual disease progressing within 5 years (3). However, a cluster of patients who are not susceptible to GTR subset exists, primarily because of tumor location. Although the low-grade biological malignancy of these tumors, products patients with both unresectable and clinically progressive disease masses receive either chemotherapy or radiotherapy, experiencing the toxicities associated with these regimens in the short and long term. The principal advances in treatment of traditional chemotherapy for LGG include carboplatin and vincristine, TPCV (thioguanine, procarbazine, lomustine, and vincristine), and weekly vinblastine monotherapy (191, 192). These conventional chemotherapeutic approaches used in pLGG patients are associated with side effects such as myelosuppression, alopecia, and less frequently ototoxicity (carboplatin) and decreased fertility potential (procarbazine) (193, 194). In addition, bevacizumab is another promising approach as it has shown improvements in the treatment of OPGs (195). Radiotherapy, a historical standard of care and time-tested efficacious therapy, has long been abandoned as a primary treatment for pLGGs. Radiation-induced late effects can be particularly devastating and include vasculopathy, stroke, endocrinopathy, cognitive impairment, and secondary malignancies (196). The decision to avoid radiotherapy in pLGG is that progression of disease may be of little consequence when there are multiple systemic treatment options available, OS remains excellent, and the risks of radiotherapy-associated late effects, particularly secondary malignancy, outweigh any potential benefits of improved progression-free survival (197). Young patients are mostly susceptible, in fact, the actual cut-off age for radiation therapy is moved beyond 12 years (15).

Recently, advancements in how we have gained an insight into pLGG biology have sparked a new promising treatment in the field of pediatric neuro-oncology. Multiple investigations repeatedly affirmed that the great majorities of the pLGG exhibit alterations in their drivers that are commonly found to lead to the dual activation of the MAPK pathway and to downstream mTOR pathway (76). In addition, novel technologies in NGS have allowed the discovery of additional new altered drivers, including *FGFR* (76). Following these findings, in the last few years, have been developed several drugs targeting the pLGG MAPK and mTOR pathway (79–102, 116, 138–143, 154–156).

Dabrafenib and vemurafenit were demonstrated to have an outstanding efficacy on pLGG mutant BRAF p.V600E patients in early phase clinical trials. Vemurafenib is a small competing drug that selectively recognizes the ATP-binding domain of the BRAF p.V600E mutant. It has been proven efficacious in the management of metastatic melanoma, a malignancy frequently mutated for BRAF. This drug’s function has more recently been shown to be successful in BRAF p.V600E mutated pediatric malignant astrocytomas, while less data is currently available on its use in LGG patients, of which there are more studies on the combination of dabrafenib and trametinib (**Table 3**) (88, 106, 198–200, 205). The efficacy of the vemurafenib treatment in our small cohort of patients affected by pLGG is promising, with a rate of response of about 60% (88).

**Table 3:** List of results of targeted therapy in pLGG.

Study	N Patients	Additional information on patient population	Results	Reference
Children with LGGs and treated with vemurafenib	n=7	BRAF p.V600E	1 CR, 3 PR, 1 SD, 1 PD. In addition, in 1 patient, the follow-up is too short to establish the clinical response.	Del Bufalo et al., 2018 (88)
Children with recurrent or progressive brain tumors treated with vemurafenib	n=19	BRAF p.V600E	1 CR, 5 PR and 13 SD	Nicolaides et al., 2020 (198)
Children with pLGGs or PHGGs treated with dabrafenib or vemurafenib	n=67	56 of 67 pts have pLGGs and carried BRAF p.V600E	80% of pLGGs with BRAFi had a OS 3-year PFS was 49.6% in pLGGs with BRAFi vs 29.8% treated with CV	Nobre et al., 2020 (199)
Children with LGGs or plexiform neurofibroma with refractory tumor treated with trametinib.	n= 105	60 pts with PLGG and 45 pts with PN	53 pts with PLGG were evaluable. 1 CR, 7 PR, 17 minor response (MR), 23 SD and 5 PD	Perreault et al., 2022 (100)
Children with recurrent/progressive LGGs treated with trametinib	n= 10	4 pts carried KIAA1549-BRAF fusion, 4 pts carried NF1 mutation, 1 pt carried FGFR mutation and 1 pts carried CDKN2A loss	2 PR, 2 MR and 6 SD	Manoharan et al., 2020 (167)
Children with sporadic PA treated with trametinib	n=6	5 pts carried KIAA1549-BRAF fusion; 1 carried hotspot FGFR1/NF1/PTPN11 mut	2 PR, 3 MR	Kondyli et al., 2018 (168)
Children with progressive LGGs treated with trametinib	n=18	8 KIAA1549:BRAF-fusions, 3 NF1 alterations, 1 BRAF V600E mutation and 1 FGFR1 K654Q mutation, 5 not detected	6 PR, 2 MR and 10 SD as best OR. DCR was 100% under therapy. Responses were observed in KIAA1549:BRAF- as well as neurofibromatosis type 1 (NF1)-driven tumors. PD was observed in 3 pts after interruption of trametinib.	Selt et al., 2020 (171)
Children with LGGs treated with trametinib or dabrafenib plus trametinib	n=139	91 pts carried BRAF p.V600 mut and treated with trametinib; 48 pts treated with dabrafenib + trametinib	In 47 pts with pLGGs ORR were 15% (trametinib) vs 25% (dabrafenib plus trametinib).	Bouffet et al., 2023 (107)
Children with LGGs treated with drabrafenib plus trametinib	N=110	73 pts carried BRAF p.V600 mut and treated with D+T and 37 pts treated with CV	ORR (CR+PR) was 47% with D+T vs 11% with CV. 12-mo PFS were 67% D+T vs 26% CV	Bouffet et al., 2022 (170)
Children with LGGs treated with drabafenib with trametinib	n=110	73 pts carried BRAF pV600E and treated with D+T vs 37 pts treated with CV	ORR was 46.6% in pts treated with D+T vs 10.8% with CV DOR was 23.7 months (D+T) vs not estimable (CV) PFS was 20.1 months(D+T) vs 7.4 months (CV)	FDA (200)
Children with BRAF aberration or NF1 associated recurrent, refractory, or progressive LGG treated with Selumetinib	n=50	25 PA pts with BRAF aberration and 25 pts with NF1 associated with pLGG	36% of PS patients had a sustained PR vs 40% of NF1 pts	Fangusaro et al., 2019 (165)
Children with recurrent optic pathway and hypothalamic low-grade glioma without NF1 treated with selumetinib	n=25	BRAF p.V600E or KIAA1549-BRAF fusion	6 pts (24%) had PR, 14 (56%) had SD and 5 (20%) PD 2-y PFS was 78 ± 8.5%. 19 pts were evaluable for visual acuity: which improved in 4 pts 21%, was stable in 13 68% and worsened in 11%. 26% had improved visual fields and 74% were stable.	Fangusaro et al., 2021 (181)
Children with recurrent or refractory LGG treated with selumetinib	N=66	25 pts with non-NF-1 and non-optic pathway recurrent/refractory PA; 25 pts with NF-1-associated LGG; 16 pts with non-NF-1 optic pathway/hypothalamic LGG	5 (32%) pts with non-NF-1 and non-optic pathway recurrent/refractory PA carried BRAF aberrations had PR with 2-year PFS (66+/-11%). 10 (40%) pts with NF-1-associated LGG had PR (2-y PFS of 96+/-4%). 2 (12.5%) pts with non-NF-1 optic pathway/hypothalamic LGG had a PR (2-y PFS of 65+/-13%).	Fangusaro et al., 2017 (201)
Pediatric patients with non-NF1-associated, non- OPG and non-pilocytic recurrent/progressive LGG, treated with selumetinib	n= 23	LGG carried BRAF p.V600E or BRAF-KIAA1549 fusion 13 tumours with BRAF fusion and 11with BRAF p.V600E	5 pts (22%) with PR, 12 (52%) with SD and 6 (26%) had PD with a 2-year PFS of 75 + 9%.	Fangusaro et al., 2022 (202)
Children with LGGs treated with everolimus	n=10	mTOR-pmTOR pathway overexpression	SD in 7 patients, PR in 1 and PD in 2 patients.	Cacchione et al., 2021 (190)
Children with recurrent and provessive LGG treated with everolimus	n=65	BRAF alteration in 36/65 pts	PFS is 63% for total cohort; PFS is 64% for the activated and 61% for the non-activated PI3K/Akt/mTOR pathway pts. In 52 pts the central imaging review revealed 1 PR, 1 CR, 33 SD and 17 progressive disease at the end of study therapy.	Mueller et al., 2020 (203)
Children with recurrent, radiographically progressive LGGs and treated with everolimus	n=23	/	2 PR, 10 SD without CR, 11 PD 2-y PFS was 39 ± 11%, 3-y PFS was 26 ± 11%, and 5-year PFS was 26 ± 11%; 2 pts died of disease. The 2-y, 3-y and 5-y OS were all 93 ± 6%.	Wright et al., 2021 (204)
Children with pLGGs treated with dabrafenib, everolimus, trametinib and vemurafenib.	n=55	dabrafenib (n=15), everolimus (n=26), trametinib (n=11), vemurafenib (n=3).	EFS from targeting therapy initiation were: 62.1% for 1-year EFS 38.2% for 3-year EFS 31.8% for 5-year EFS	Tsai et al., 2022 (205)

Furthermore, *BRAF*-fusions in pLGGs drive resistance/escape mechanisms to targeted inhibitors. For example, *KIAA1549-BRAF* has innate resistence to first-generation BRAFi vemurafenib as well as paradoxically triggered by PLX4720 treatment resulting in faster growth of tumor (90), while it shows a strong response to clinically available MEKi (e.g., trametinib) (206). Several studies showed that trametinib seems a appropriate choice in refractory as well as in progressive pLGG with *KIAA1549-BRAF* fusion and suggested that warrants further investigations in case of progression (**Table 3**) (167, 168, 171). The data of the study on progressive pLGGs lend weight to the class MEKi efficacy in pLGGs and the necessity of a randomized upfront trial of trametinib over current chemotherapy standard regimens (171). A phase 2 trial on patients with refractory/progressing LGG (NF1 patients and patients who carried KIA11549-BRAF fusion) and treated with trametinib, will investigate the molecular biological mechanisms that drive tumor development and progression, and the involvement of these mechanisms in resistance to therapy (100). Bouffet et al. showed the results of a phase II trial in which was compared the ORR in patients with pLGG who carried BRAF p.V600E mutation treated with both dabrafenib and trametinib (47%) or standard chemotherapy (CV) treatment (11%) (170).

In addition, in a cohort of both children and young adults treated for refractory tumors that have mutations or fusions resulting in activation of the MAPK pathway showed restricted selumetinib efficacy, suggesting that the mutation status of the pathway alone is sufficient to provide a predictor of the response to monotherapy with selumetinib for those tumors (207). In contrast, a phase II clinical trial on selumetinib among pediatric patients with relapsed and refractory LGG demonstrated impressive outcomes in sporadic OPG and hypothalamic LGG patients, with 24% partial response rate and 56% of patients showing long-term stability (104). In **Table 3** are showed results of selumetinib on pLGG (165, 181, 201, 202).

With the discovery that many relapsed/refractory pLGGs have activation of mTOR pathway more treatment options may be possible for patients, including everolimus, a brain-penetrant drug already approved by the FDA for the treatment of SEGA in children (**Table 3**) (184, 190, 203, 204, 208–210). In our published experience, everolimus is a feasible treatment for p.V600E *wild-type* non-TSC pLGG patients (210). Interestingly, everolimus has been shown to synergize with carboplatin in preclinical models *in vitro* and *in vivo* by suppressing the conversion of glutamine and glutamate into glutathione (211). The PI3K-AKT-mTOR signaling cascade has been considered the major escape mechanism for BRAF-fusion. Jain and colleagues have shown that combinatorial targeting using MEKi and mTORi for *BRAF*-fusion-driven tumors is effective in overcoming such emergent resistance to single-agent therapy, highlighting preclinical rationales for using MEKi and mTORi. Very limited experience exists for combination therapies. However, in *BRAF* WT cells, everolimus and AZD6244 (MEK1/2 inhibitor) have proven to be superior compared to respective monotherapies (212).

To date, due to the results obtained from the various trials, the Children’s Oncology is investigating the possibility of the first-line treatment with MEKi, both as a single agent and in combination with chemotherapy in children with pLGG and relapsed cases.

Currently, the combination therapy development for pLGG patients is under investigation, and in particular, to date, the benefits of personalized therapies based on the administration of a single drug or with multiple combination drugs in non-resectable pLGG patients are unknown. In pLGGs patients, PNOC021 is the first study evaluating the combination of an mTORi (everolimus) and a MEKi (trametinib) to see if there is a possibility of achieving a safe MTD for this combination therapy strategy.

Tergeted drugs have less side systemic effects, however their fairly recent use precludes yet a comprehensive characterization regarding their long-term effects. The majority of short-term adverse events of targeted therapies are temporary and easily manageable, including creatine phosphokinase (CPK) elevation, cutaneous, cardiologic and ocular sequence and toxicities (164, 213). Long-term impact of BRAF, MEK, and mTOR inhibitors on mental and growth consequence in children remain uncertain due to the short follow up described to date. Of note, a limitation of target therapy remains the rebound effect of tumor growth at treatment suspension.

## 4 Conclusion

This literature review shows that targeted therapy is a feasible approach for pLGGs. Advances in cancer therapies including chemotherapy, radiation therapy, and surgery have significantly improved cancer treatment and outcomes for patients. However, these treatments can lead to a number of toxicities, which are related to a negative impact on their long-term health as well as quality of life. A greater understanding of tumor biology and a germline and somatic genomic approach will play a central role in the therapy strategy of pLGG for the development of increasingly tailored therapies. Limitations still exist regarding the adverse effects of long-term treatment.

## Author contributions

AM and AC conceptualized the work. SC, GB and FF wrote the manuscript. AM and AC contributed to the finishing of the work and revised it critically for important intellectual content. All authors finally approved the version to be published and agreed to be accountable for all aspects of the work in ensuring that questions related to the accuracy or integrity of any part of the work are appropriately investigated and resolved.
